# A Rare Case of Shewanella putrefaciens Bacteremia Due to Cellulitis in a Patient With Chronic Lymphedema Without Marine Exposure

**DOI:** 10.7759/cureus.100421

**Published:** 2025-12-30

**Authors:** Owais Gul, Fawad Talat, Frank Mulindwa, Hadia Waheed, Saqib Gul

**Affiliations:** 1 Internal Medicine, United Health Services Wilson Medical Center, Johnson City, USA; 2 Internal Medicine, Hamdard College of Medicine and Dentistry, Karachi, PAK

**Keywords:** antibiotics, cellulitis, gram-negative bacteremia, septic shock, shewanella putrafaciens

## Abstract

*Shewanella putrefaciens (S. putrefaciens)* is a Gram-negative anaerobic organism responsible for opportunistic infections in immunocompromised hosts. It can cause a wide range of infections in humans, including skin and soft tissue infections (SSTIs) and bacteremia. Peripheral vascular disease and the presence of chronic bilateral lower extremity ulcers may serve as risk factors for these infections. This is a case report of an 80-year-old male with a history of peripheral vascular disease and chronic lymphedema of the bilateral lower extremities who presented with generalized weakness. He was found to have septic shock and Shewanella putrefaciens bacteremia secondary to left lower extremity cellulitis. There was no history of exposure to the marine environment. However, the patient had multiple comorbid conditions and chronic denuded skin due to lymphedema, which may have provided a nidus for bacterial proliferation and entry into the bloodstream.

## Introduction

Cellulitis is a deep skin infection typically caused by Gram-positive organisms, such as *Staphylococcus aureus* and *Streptococcus pyogenes*; Gram-negative organisms are less common and mainly affect immunocompromised individuals [1]. *Shewanella* is a group of Gram-negative, rod-shaped, facultative anaerobic microorganisms that can lead to opportunistic infections in immunocompromised hosts. Of approximately 30 *Shewanella* strains, *Shewanella algae (S. algae)* and *S. putrefaciens* are most commonly implicated in human infections, often associated with exposure to marine water or the consumption of seafood. *S. putrefaciens*, a rare pathogen first identified in 1931, mainly causes skin, soft tissue, or intra-abdominal infections, but can also lead to serious infections, such as pneumonia in ventilated patients and bloodstream infections [2]. Pediatric infections have also been reported, including neonatal sepsis. Complications from skin and soft tissue infections (SSTIs), such as necrotizing fasciitis, Fournier's gangrene, arthritis, and osteomyelitis, have also been reported. Mortality from sepsis and septic shock has been reported to be up to 20% in one study [3]. We present a case of *S. putrefaciens* causing bacteremia and cellulitis in an 80-year-old male with multiple comorbidities and no history of marine exposure.

## Case presentation

**Figure 1:**
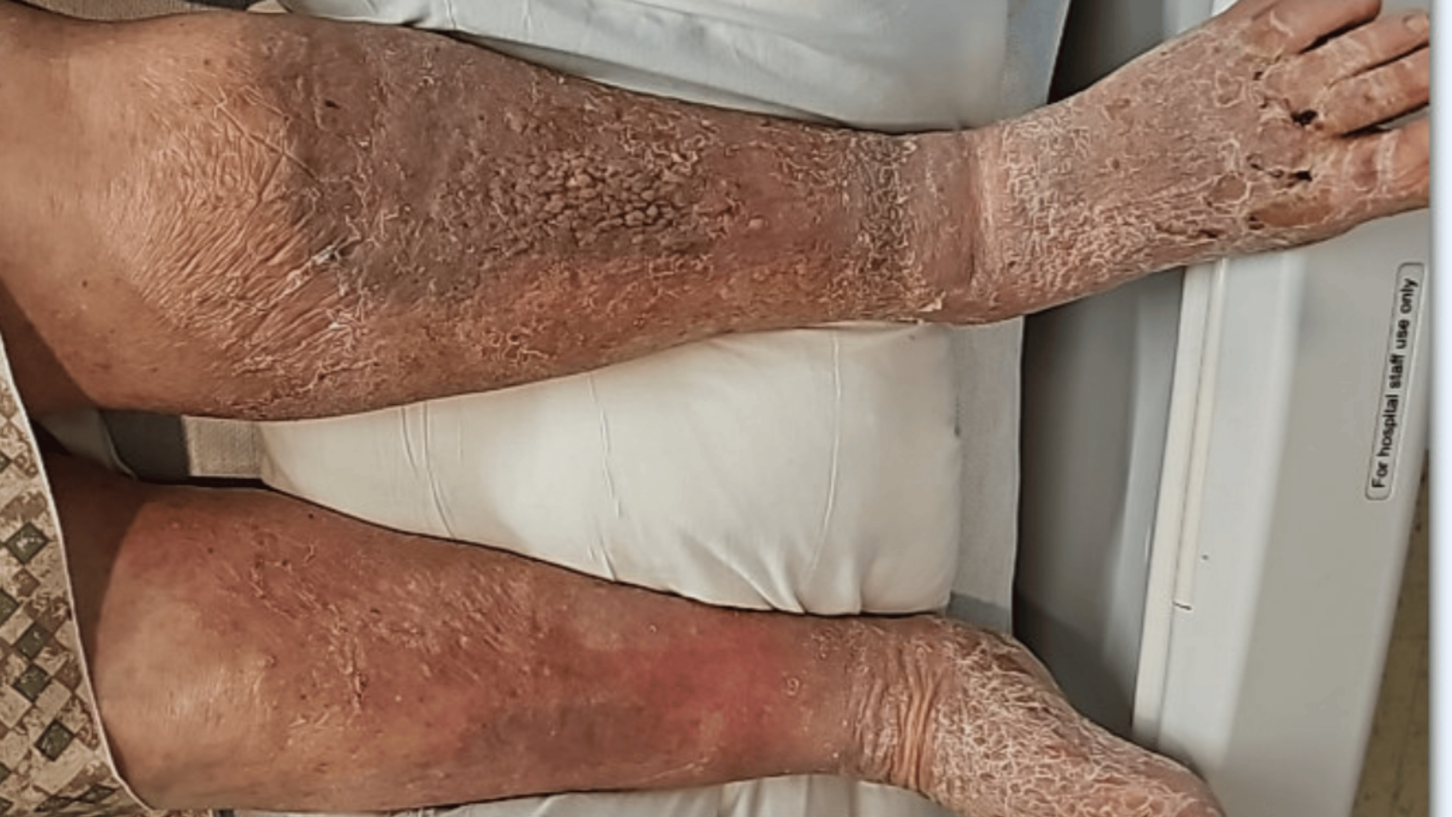
Chronic bilateral lower limb lymphedema, with greater swelling on the left side than the right

An 80-year-old male with a past medical history of stage 1 colon cancer status post hemicolectomy and currently in remission, peripheral vascular disease, and chronic bilateral lower extremity lymphedema presented to the emergency department with a chief complaint of generalized weakness for the past two weeks. The patient had been unable to get out of bed for the past two days. The review of systems was also positive for worsening pain and swelling in the left leg associated with chills, malaise, and body aches. He had no history of contact with seawater, fish, or the marine environment. On presentation, he was found to be hypothermic with a body temperature of 94.5 °F and hypotensive with a blood pressure of 88/50 mmHg. On examination, bilateral lymphedema of the lower limbs was noted. Additionally, his left leg was more swollen, warm, and tender than the right one. Denuded skin was observed on the bilateral lower extremities. However, there was no open wound or purulence identified, so wound cultures could not be obtained (Figure 1).

Doppler ultrasound of the bilateral lower limbs ruled out deep venous thrombosis. Initial laboratory analysis revealed significantly increased values for white blood cell count (WBC), C-reactive protein (CRP), serum lactic acid, serum creatinine, and serum blood urea nitrogen (BUN), along with the presence of moderate hyponatremia (Table 1).

**Table 1:** Laboratory analysis with comparison between patient's initial labs and follow-up labs

Parameters	Initial labs	Follow-up labs	Reference ranges
White blood cell count (WBC), x 10^3 ^cells/µL	37.9	9.5	4-11
Lactic acid, mmol/L	3.3	1.9	<2.0
C-reactive protein, mg/dL	7.9	1.5	<1.0
Creatinine, mg/dL	2.0	0.9	<1.3
Blood urea nitrogen (BUN), mg/dL	85	27	<24
Serum sodium, mEq/L	127	136	135-145

**Figure 2:**
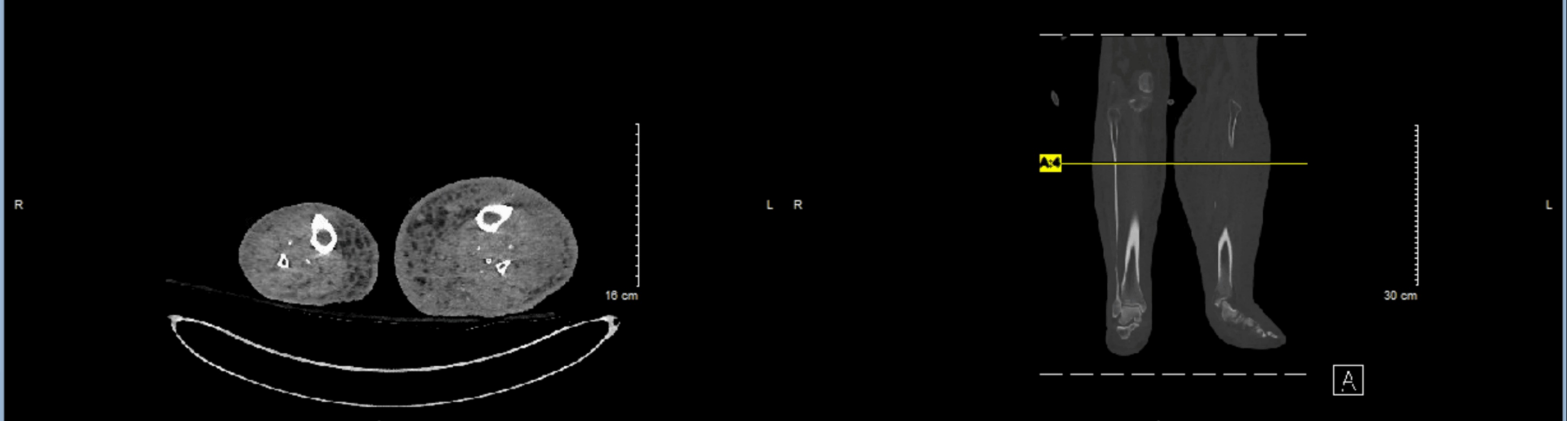
CT scan without contrast of bilateral extremities also showing greater soft tissue edema on the left compared with the right. CT: computed tomography

Two sets of blood cultures were collected, and Gram stains quickly demonstrated the growth of Gram-negative bacilli. The source of bacteremia was likely left leg cellulitis, as an extensive workup, including a CT scan of the chest, abdomen, and pelvis, as well as a urinalysis, did not reveal any other source of infection. In this context, transthoracic and transesophageal echocardiograms were performed to rule out infective endocarditis, and these studies did not show any evidence of valvular vegetations. A CT scan of the bilateral lower extremities without intravenous contrast was obtained, which showed greater soft tissue edema in the left leg than the right, with no soft tissue air or gas present, thereby ruling out necrotizing fasciitis. Additionally, no deep tissue abscess was seen. However, the study was limited by the lack of intravenous contrast, which was contraindicated due to impaired renal function (Figure 2).

Management was initiated for septic shock presumed secondary to left leg cellulitis. The patient was given intravenous fluid boluses and was subsequently started on vasopressor support because of persistent hypotension. He was also started on broad-spectrum intravenous antibiotic therapy, including cefepime 2 g every eight hours, vancomycin with dosing based on creatinine clearance, and metronidazole 500 mg every eight hours. His blood pressure gradually improved, and his serum lactic acid, WBC count, CRP, BUN, and creatinine levels began to trend downward on follow-up laboratory testing, along with resolution of hyponatremia (Table 1). After five days, the initial blood cultures with Gram stain analysis were finalized and demonstrated growth of *S. putrefaciens*, which was pan-sensitive to cephalosporins, fluoroquinolones, gentamicin, and piperacillin-tazobactam (Table 2).

**Table 2:** Initial blood culture and susceptibility report showing pan-sensitivity of Shewanella putrefaciens towards the antibiotics

Antibiotic	Minimal inhibitory concentration (MIC), µg/mL	Interpretation
Cefepime	<2	Susceptible
Ciprofloxacin	<1	Susceptible
Gentamicin	<4	Susceptible
Levofloxacin	<2	Susceptible
Piperacillin + tazobactam	<16	Susceptible
Trimethoprim/sulfamethoxazole	<2/38	Susceptible

The patient's blood cultures were repeated 48 hours after the initial set and were negative. His clinical condition continued to improve, and his cellulitis appeared to be resolving. The infectious disease team was consulted and recommended de-escalating antibiotics to intravenous cefepime alone for a total of 14 days from the date of the negative blood culture. The patient was eventually discharged home with the plan to complete the remainder of the antibiotic course at home following placement of a peripherally inserted central catheter (PICC). He followed up with his primary care provider and the infectious disease team two weeks after completing antibiotics. Fortunately, his cellulitis continued to improve, and he experienced no adverse effects from long-term antibiotic therapy.

## Discussion

As rare opportunistic pathogens, *Shewanella* species can cause a wide range of diseases in humans. These include SSTIs, as well as invasive conditions such as sepsis, hepatobiliary infections, peritonitis, and otitis media. However, bacteremia caused by *S. putrefaciens* is uncommon, with only a limited number of cases reported in the literature. *S. putrefaciens* has been associated with several predisposing factors. In addition to immunosuppression, diabetes mellitus, peripheral vascular disease, chronic lower extremity ulcers, low socioeconomic status, and poor personal hygiene have been identified as risk factors [3]. In our case, the predisposing factors were denuded skin due to lymphedema and peripheral vascular disease. Although bacteremia secondary to cellulitis is rare, poor personal hygiene in the setting of impaired skin integrity may have facilitated bacterial entry into the bloodstream. Chronic lower limb ulcers have also been described as a potential portal of entry for bacteria [3].

The exact pathophysiological mechanisms and virulence factors by which *S. putrefaciens* causes infections have not been fully elucidated. However, current evidence suggests that *Shewanella* subspecies tend to colonize susceptible tissues and can subsequently lead to local and, in predisposed patients, invasive infections [3,4]. There are reports of respiratory colonization by this organism resulting in lower respiratory tract infections. *S. putrefaciens* can be present in sputum as part of mixed flora or as a contaminant, which can obscure the clinical significance of its detection [5,6]. In our patient, however, the combination of positive blood cultures with Gram stain, clinical signs and symptoms, and laboratory evidence of septic shock underscores the pathogenic role of this rare organism.

There have been a few reported cases of infective endocarditis caused by *S. putrefaciens* in the medical literature [7]. In our case, transthoracic and transesophageal echocardiograms excluded the presence of valvular vegetations. *S. putrefaciens* is generally susceptible to aminoglycosides, carbapenems, erythromycin, and quinolones; however, resistance to penicillin is well documented [8]. Susceptibility to ampicillin and cephalosporins can be variable, with isolates showing greater sensitivity to third- and fourth-generation cephalosporins compared with first- and second-generation cephalosporins [9]. The isolate identified in our case was susceptible to aminoglycosides, quinolones, and cephalosporins. Therefore, the patient was treated with intravenous cefepime, a fourth-generation cephalosporin, for a total of 14 days starting from the date of the first negative blood culture.

This report highlights the importance of recognizing rare infectious pathogens such as *S. putrefaciens*, even in the absence of obvious risk factors like marine exposure, as seen in our case. Early identification of such pathogens can guide timely management and promote faster patient recovery.

## Conclusions

Cellulitis is primarily caused by Gram-positive organisms, and Gram-negative infections are uncommon. *S. putrefaciens*, a Gram-negative organism, can cause SSTIs in humans, which may progress to bacteremia. Immunocompromised individuals are at risk, but peripheral vascular disease and impaired skin integrity due to chronic lymphedema can create a medium for bacterial colonization and bloodstream entry, which may have occurred in our patient. Additionally, exposure to the marine environment is a known risk factor for infection with this organism, which was not present in our case. Treatment involves the use of broad-spectrum antibiotics, but ultimately depends on the culture results and susceptibility of the isolated organism. This was the approach in our patient, who was treated with intravenous cefepime for a total of 14 days after the initial blood culture demonstrated pan-sensitivity to cephalosporins. The report underscores the importance of timely diagnosis of rare infectious pathogens to facilitate early patient recovery.

## References

[1] Gunderson CG, Martinello RA (2012). A systematic review of bacteremias in cellulitis and erysipelas. J Infect.

[2] Yu K, Huang Z, Xiao Y, Wang D (2022). Shewanella infection in humans: Epidemiology, clinical features and pathogenicity. Virulence.

[3] Müller S, von Bonin S, Schneider R, Krüger M, Quick S, Schröttner P (2022). Shewanella putrefaciens, a rare human pathogen: a review from a clinical perspective. Front Cell Infect Microbiol.

[4] Oh HS, Kum KA, Kim EC, Lee HJ, Choe KW, Oh MD (2008). Outbreak of Shewanella algae and Shewanella putrefaciens infections caused by a shared measuring cup in a general surgery unit in Korea. Infect Control Hosp Epidemiol.

[5] Basir N, Yong AM, Chong VH (2012). Shewanella putrefaciens, a rare cause of splenic abscess. J Microbiol Immunol Infect.

[6] Ullah S, Mehmood H, Pervin N, Zeb H, Kamal KR, Liaqat S (2018). Shewanella putrefaciens: an emerging cause of nosocomial pneumonia. J Investig Med High Impact Case Rep.

[7] Dhawan B, Chaudhry R, Mishra BM, Agarwal R (1998). Isolation of Shewanella putrefaciens from a rheumatic heart disease patient with infective endocarditis. J Clin Microbiol.

[8] Benaissa E, Abassor T, Oucharqui S, Maleb A, Elouennass M (2021). Shewanella putrefaciens: a cause of bacteremia not to neglect. IDCases.

[9] Holt HM, Gahrn-Hansen B, Bruun B (2005). Shewanella algae and Shewanella putrefaciens: clinical and microbiological characteristics. Clin Microbiol Infect.

